# Maresin 1 activates LGR6 receptor promoting phagocyte immunoresolvent functions

**DOI:** 10.1172/JCI168084

**Published:** 2023-01-17

**Authors:** Nan Chiang, Stephania Libreros, Paul C. Norris, Xavier de la Rosa, Charles N. Serhan

Original citation: *J Clin Invest*. 2019;129(12):5294–5311. https://doi.org/10.1172/JCI129448

Citation for this corrigendum: *J Clin Invest*. 2023;133(2):e168084. https://doi.org/10.1172/JCI168084

The authors recently became aware that representative illustrations presented in Supplemental Figure 7A might be mistaken for original data. As this schematic was used strictly for demonstrative purposes, it has been removed from the figure for clarity. A tabular representation of the experimental findings is provided in Supplemental Table 3. The supplemental document has been updated online.

## Version 1 01/17/2023

Electronic publication

